# Sperm-contributed centrioles segregate stochastically into blastomeres of 4-cell stage *Caenorhabditis elegans* embryos

**DOI:** 10.1093/genetics/iyad048

**Published:** 2023-03-29

**Authors:** Pierre Gönczy, Fernando R Balestra

**Affiliations:** Swiss Institute for Experimental Cancer Research (ISREC), School of Life Sciences, Swiss Federal Institute of Technology Lausanne (EPFL), Lausanne CH-1015, Switzerland; Swiss Institute for Experimental Cancer Research (ISREC), School of Life Sciences, Swiss Federal Institute of Technology Lausanne (EPFL), Lausanne CH-1015, Switzerland; Departamento de Genética, Universidad de Sevilla, Sevilla 41080, Spain; Centro Andaluz de Biología Molecular y Medicina Regenerativa-CABIMER, Universidad de Sevilla-CSIC-Universidad Pablo de Olavide, Sevilla 41092, Spain

**Keywords:** *Caenorhabditis elegans*, sperm, centriole, early embryo, *zyg-1*

## Abstract

Whereas both sperm and egg contribute nuclear genetic material to the zygote in metazoan organisms, the inheritance of other cellular constituents is unequal between the 2 gametes. Thus, 2 copies of the centriole are contributed solely by the sperm to the zygote in most species. Centrioles can have a stereotyped distribution in some asymmetric divisions, but whether sperm-contributed centrioles are distributed in a stereotyped manner in the resulting embryo is not known. Here, we address this question in *Caenorhabditis elegans* using marked mating experiments, whereby the presence of the 2 sperm-contributed centrioles is monitored in the embryo using the stable centriolar component SAS-4::GFP, as well as GFP::SAS-7. Our analysis demonstrates that the distribution of sperm-contributed centrioles is stochastic in 4-cell stage embryos. Moreover, using sperm from *zyg-1* mutant males that harbor a single centriole, we show that the older sperm-contributed centriole is likewise distributed stochastically in the resulting embryo. Overall, we conclude that, in contrast to the situation during some asymmetric cell divisions, centrioles contributed by the male germ line are distributed stochastically in embryos of *C. elegans*.

## Teaser

Here, Gönczy and Balestra explore the question of sperm-contributed centriole distribution in early *C. elegans* embryos, finding a stochastic distribution of sperm-contributed centriole at the 4-cell stage. Experiments using a genetic inactivation strategy show that the older sperm-contributed centriole is likewise stochastically distributed. The authors conclude that centrioles contributed by the male germline are distributed stochastically in embryos of *C. elegans*, in contrast to what is observed during some asymmetric cell divisons.

## Introduction

Whereas the genetic material is contributed in an equal manner to the zygote by the 2 parental gametes of metazoan organisms, this is not the case for the remainder of the cellular material. In most species, the egg contributes the bulk of the messenger RNAs, proteins, and cytoplasmic constituents to the zygote. The same holds true for mitochondria, which are inherited and retained from maternal stores by the developing embryo. Conversely, the sperm is the sole contributor of centrioles, which are absent from the egg (reviewed by Schatten 1994; Delattre and Gönczy 2004). Such differential contribution of centrioles is critical for endowing the zygote with strictly 2 centrioles at the onset of life. Whether the 2 sperm-contributed centrioles are segregated thereafter to specific cells of the developing embryo is not known.

Centrioles are small microtubule-based organelles that are critical for fundamental cellular processes across eukaryotes (reviewed by Bornens 2012; Winey and O’Toole 2014). Centrioles are key notably in their capacity as basal bodies that seed the formation of the axoneme of cilia and flagella in specific cell types, including sperm cells of most species. Moreover, in animal systems, centrioles recruit the pericentriolar material (PCM), thus forming the centrosome, which acts as an important microtubule-organizing center. Through this role, centrioles are important for fundamental cellular processes such as polarity and division.

Centrosomes have an inherent cell generational asymmetry: most proliferating cells are born with 2 centrioles, a so-called mother centriole that has been generated at least 1 cell generation prior and a so-called daughter centriole that has been generated during the previous cell cycle (reviewed by Nigg and Stearns 2011). Toward the onset of S phase, both mother and daughter centrioles seed the formation of a procentriole in their vicinity, yielding 2 centrosomes, each containing a centriole/procentriole pair, which direct bipolar spindle assembly during mitosis.

Interestingly, centriole inheritance is stereotyped in some cases of asymmetric cell division (reviewed by Yamashita 2009). For instance, in germ line stem cells of the *Drosophila* testis, the centrosome harboring the older centriole, which is referred to as the older centrosome, is inherited systematically by the cell that retains the stem cell fate (Yamashita *et al*. 2007). Perturbing such stereotyped inheritance through mutation of the PCM protein centrosomin randomizes centrosome distribution and results in declined stem cell function (Yamashita *et al*. 2007). Conversely, the older centrosome is inherited systematically by the ganglion mother cell during asymmetric division of *Drosophila* neuroblasts, whereas stem-like neuroblasts retain the younger centrosome (Conduit and Raff 2010; Januschke *et al*. 2011). Such stereotyped asymmetric centriole inheritance is not limited to *Drosophila*. Thus, radial glial progenitors in the ventricular zone of the developing mouse cortex also exhibit stereotyped centrosome inheritance (Wang *et al*. 2009). In this case, the mother centriole is inherited systematically by the radial glial progenitor stem cell during asymmetric division, reminiscent of the situation in the fly testis. Moreover, depletion of the mother centriole-specific protein Ninein, which is essential for mother centriole anchoring to the plasma membrane, randomizes centrosome distribution in this setting. Importantly, this is accompanied by premature depletion of radial glial progenitor from the ventricular zone (Wang *et al*. 2009).

In contrast to the wealth of information regarding the inheritance pattern of centrosomes and its importance in stem cell systems, little is known regarding the distribution of the 2 sperm-contributed centrioles in the resulting developing embryos. Conceivably, such inheritance could be stereotyped as well and thereby potentially endow specific embryonic blastomeres with paternally derived components. This paucity of information stems notably from the fact that centrioles assembled in the zygote using maternal components can be difficult to distinguish from those contributed by sperm. This experimental limitation can be circumvented with so-called marked mating experiments, which have been deployed in *Caenorhabditis elegans* and which are conceptually analogous to experiments in mammalian cells that revealed the mode of centriole duplication (Kochanski and Borisy 1990). In marked mating experiments in the worm (Kirkham *et al*. 2003; Leidel and Gönczy 2003; Balestra *et al*. 2015), a stable centriolar component is labeled in sperm with a fluorescent protein such as GFP. Upon mating with an egg devoid of this GFP-tagged centriolar component, the 2 sperm-contributed centrioles stably harbor GFP, whereas all centrioles generated in the zygote do not. Therefore, this experimental setting offers an optimal means to monitor the fate of paternally contributed centrioles in early embryos.

## Results

### Experimental design

**Fig. 1.**
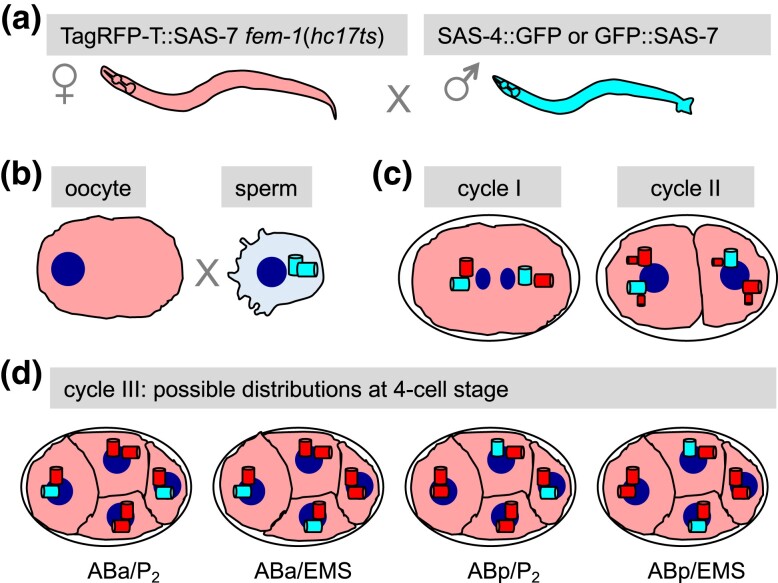
Experimental design. a and b) *fem-1*(*hc17ts*) hermaphrodites expressing TagRFP-T::SAS-7 (represented in red, left) raised at 25°C lack sperm. Such feminized hermaphrodites (hence the female gender symbol) are mated to males expressing SAS-4::GFP or GFP::SAS-7 (both represented in cyan, right); the fate of GFP-marked sperm-contributed centrioles is analyzed in the resulting embryos. c) At the end of the first cell cycle (left), a bipolar spindle assembles, with each spindle pole harboring 1 sperm-contributed centriole (cyan) and 1 procentriole assembled in the zygote with maternally contributed components (red). At the onset of the second cell cycle (right), each of the 4 centrioles seeds the formation of a procentriole in its vicinity (red). d) Possible distributions of sperm-contributed centrioles in the third cell cycle (4-cell stage embryos): ABa and P_2_, ABa and EMS, ABp and P_2_, or ABp and EMS. For simplicity, procentrioles forming in the vicinity of each centriole are not represented at the 4-cell stage.

We set out to use marked mating experiments to test whether sperm-contributed centrioles are distributed in early *C. elegans* embryos in a stereotyped fashion, or instead stochastically, focusing our analysis on the 4-cell stage. The experimental strategy is summarized schematically in Fig. 1. Hermaphrodites expressing TagRFP-T::SAS-7 and homozygous for *fem-1(hc17ts)* are raised at 25°C, which results in them lacking sperm. Such feminized hermaphrodites are then mated to males expressing endogenously tagged SAS-4::GFP or GFP::SAS-7 (Fig. 1a). As a result, the 2 sperm-contributed centrioles are marked with GFP (Fig. 1b), and each such centriole seeds the formation of 1 procentriole in its vicinity during the first cell cycle in the zygote using maternal-contributing components, since zygotic transcription begins only later. The 2 resulting centrosomes, each with a centriole/procentriole pair, constitute the 2 poles of the mitotic spindle at the end of the first cell cycle (Fig. 1c, left). Each resulting daughter cell, termed AB and P_1_, respectively, will necessarily inherit 1 of the 2 sperm-contributed centrioles (Fig. 1c, right). At the onset of the second cell cycle, each of the 4 centrioles that are present at that time seeds the formation of 1 procentriole in its vicinity (Fig. 1c, right). As illustrated in Fig. 1d, this could in principle yield 4 distinct distributions of sperm-contributed centrioles at the following cell cycle, in 4-cell stage embryos: ABa and P_2_, ABa and EMS, ABp and P_2_, or ABp and EMS.

### Stochastic distribution of sperm-contributed centrioles at the 4-cell stage

**Fig. 2.**
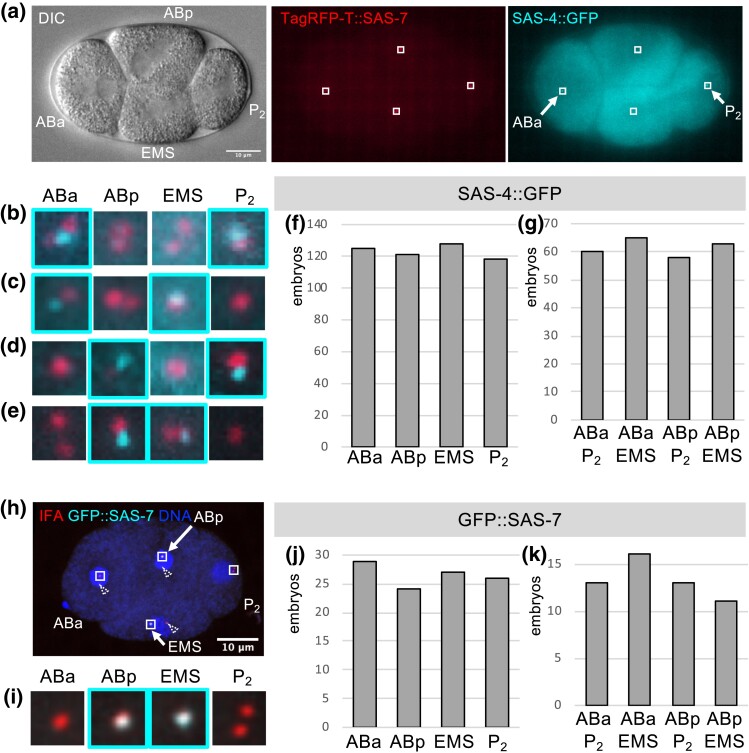
Stochastic distribution of sperm-contributed centrioles in 4-cell stage embryos. a) DIC (left), TagRFP-T::SAS-7 (center, red), and SAS-4::GFP (right, cyan) live imaging of 4-cell stage embryo resulting from the mating of TagRFP-T::SAS-7 *fem-1*(*hc17ts*) hermaphrodites raised at 25°C with males expressing SAS-4::GFP. The ABa, ABp, P_2_, and EMS blastomeres are indicated in the DIC image. Small boxes indicate insets magnified in (b). Arrows point to the ABa and P_2_ blastomeres, which inherited the 2 sperm-contributed centrioles in this particular embryo. b) Dual color (TagRFP-T::SAS-7, red; SAS-4::GFP, cyan) magnified insets of 4 regions indicated in (a); note that SAS-4::GFP is present in ABa and P_2_, as emphasized by the cyan contours. c–e) Analogous insets from embryos shown in Supplementary Fig. 1b–d, with SAS-4::GFP present in ABa and EMS (c), ABp and P_2_ (d), or ABp and EMS (e). f and g) Distribution of sperm-contributed centrioles marked by SAS-4::GFP in 4-cell stage embryos. *n* = 246 embryos, 224 analyzed by live imaging, 20 following ethanol fixation, 2 after immunofluorescence analysis. Note that 14/246 embryos were fertilized by *zyg-1*(*b1ts*) sperm that also contributed 2 SAS-4::GFP-marked centrioles (Materials and Methods). Overall occurrences (f): ABa, 125; ABp, 121; EMS, 128; and P_2_, 118. Overall occurrences (g): ABa and P_2_, 60; ABa and EMS, 65; ABp and P_2_, 58; ABp and EMS, 63. See Supplementary Table 1 for statistical analysis, as well as Supplementary Fig. 2a–f and Supplementary Table 2 for breakdown into experimental types. h) Confocal microscopy of 4-cell stage embryo resulting from mating of TagRFP-T::SAS-7 *fem-1*(*hc17ts*) hermaphrodites raised at 25°C with males expressing GFP::SAS-7, stained with antibodies against the centriolar marker IFA (red) and GFP (cyan); DNA in blue. Small boxes indicate insets magnified in (i). Arrows point to the ABp and EMS blastomeres, which inherited the 2 sperm-contributed centrioles in this particular embryo. Dashed arrowheads point to centrioles outside the magnified areas, some of which are poorly visible in the displayed optical slices. j and k) Distribution of sperm-contributed centrioles marked by GFP::SAS-7 in 4-cell stage embryos. *n* = 53 embryos, 3 analyzed by live imaging, 15 following ethanol fixation, 35 after immunofluorescence analysis. Overall occurrences (j): ABa, 29; ABp, 24; EMS, 27; P_2_, 26. Overall occurrences (k): ABa and P_2_, 13; ABa and EMS, 16; ABp and P_2_, 13; ABp and EMS, 11. See Supplementary Table 1 for statistical analysis, as well as Supplementary Fig. 2g–l and Supplementary Table 2 for breakdown into experimental types.

We deployed the above experimental strategy using primarily SAS-4::GFP because prior work established that this evolutionarily conserved centriolar protein does not undergo significant exchange once incorporated in the centriole (Kirkham *et al*. 2003; Leidel and Gönczy 2003; Balestra *et al*. 2015). As illustrated in Fig. 2a and Supplementary Fig. 1, the TagRFP-T::SAS-7 signal serves to identify the location of centrioles in each cell, whereas the SAS-4::GFP signal is monitored to determine the distribution of sperm-contributed centrioles. Importantly, this analysis uncovered that the fraction of 4-cell embryos harboring a sperm-contributed centriole in ABa is indistinguishable from that harboring a sperm-contributed centriole in ABp (Fig. 2b–f; Supplementary Fig. 2a–c; see Supplementary Tables 1 and 2 for statistical analysis; *n* = 246 embryos). Similarly, the fraction of 4-cell embryos with a sperm-contributed centriole in EMS is indistinguishable from that with a sperm-contributed centriole in P_2_ (Fig. 2b–f; Supplementary Fig. 2a–c; Supplementary Tables 1 and 2). Moreover, there is no stereotyped pair-wise arrangement of cells harboring sperm-contributed centrioles; instead, the fraction of embryos with sperm-contributed centrioles in ABa and P_2_ is indistinguishable from that in ABa and EMS, ABp and P_2_, or ABp and EMS (Fig. 2g; Supplementary Fig. 2d–f; Supplementary Tables 1 and 2).

To test whether this outcome is specific to SAS-4::GFP, we conducted analogous experiments using GFP::SAS-7, which exhibits a strong signal in sperm centrioles, making it a potentially suitable candidate for marked mating experiments. We found, however, that part of the GFP::SAS-7 signal present in sperm centrioles is lost shortly after fertilization, such that we relied primarily on immunofluorescence analysis in this case to more reliably detect sperm-contributed centriolar GFP::SAS-7 in 4-cell stage embryos. We used antibodies against the centriolar marker IFA to identify all centrioles, as well as against GFP to detect specifically sperm-contributed centrioles (Fig. 2h and i). This analysis demonstrated that, just like for SAS-4::GFP, the distribution of sperm-contributed centrioles is indistinguishable when comparing ABa with ABp, as well as EMS with P_2_ (Fig. 2j; Supplementary Fig. 2g–i; Supplementary Tables 1 and 2; *n* = 53 embryos). Moreover, no stereotyped pair-wise arrangement of cells harboring sperm-contributed centrioles is observed in this case either (Fig. 2k; Supplementary Fig. 2j–l; Supplementary Tables 1 and 2). Taken together, these experiments lead us to conclude that paternally contributed centrioles are distributed in a stochastic fashion in 4-cell stage *C. elegans* embryos.

### The older sperm-contributed centriole can be present in any blastomere at the 4-cell stage

We next set out to address whether there might be a preferential inheritance of the older vs the younger sperm-contributed centriole in the resulting embryos. However, no marker is known in *C. elegans* that would distinguish the older centriole from the younger one. Proteins such as Ninein that are present specifically at the mother centriole in other systems are absent from the worm genome, and the 2 sperm-contributed centrioles appear similar by electron microscopy (Wolf *et al*. 1978). To bypass this limitation, we designed a genetic strategy relying on the temperature-sensitive mutant allele *zyg-1*(*b1ts*) (Wood *et al*. 1980). ZYG-1 is a kinase essential for procentriole formation in *C. elegans* and a relative of the Polo-like-kinase PLK4 that exerts a similar function in other metazoan organisms (O’Connell *et al*. 2001). The *zyg-1*(*b1ts*) mutant allele is endowed with normal function at the permissive temperature of 15°C but exhibits a strong and perhaps complete loss of function phenotype at the restrictive temperature of 25°C (Wood *et al*. 1980; O’Connell *et al*. 2001).

**Fig. 3.**
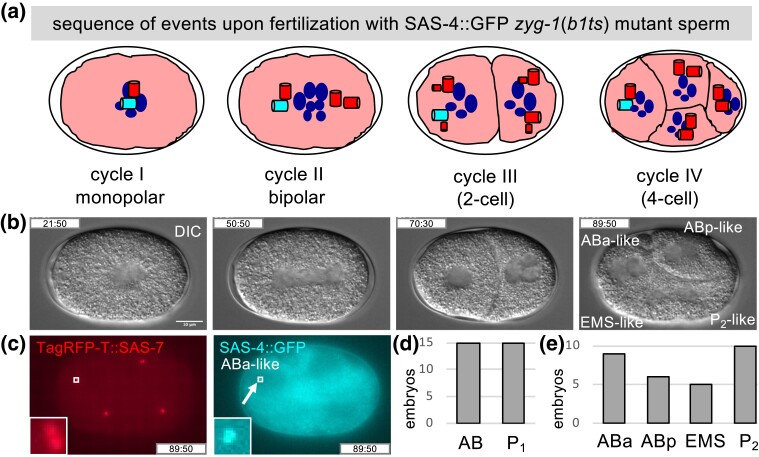
The older sperm-contributed centriole can be present in any blastomere at the 4-cell stage. a) Schematic of sequence of events in embryos following fertilization of *fem-1*(*hc17ts*) hermaphrodites expressing TagRFP-T::SAS-7, raised at 25°C and thus lacking sperm, by *zyg-1*(*b1ts*) mutant males shifted to 25°C during spermatogenesis, leading to sperm with only 1 centriole marked by SAS-4::GFP. During the first cell cycle, this single centriole seeds the formation of a procentriole in its vicinity, but the first division is monopolar, leading to aberrant chromosome segregation and cleavage failure (left). In the second cell cycle, each centriole seeds the formation of a procentriole, such that a bipolar spindle assembles during mitosis (second panel), generating AB-like and P_1_-like cells in the third cell cycle (third panel). These cells divide asynchronously, with the AB-like cell going first, yielding embryos at the fourth cell cycle that harbor AB-like, ABp-like, EMS-like, and P_2_-like blastomeres (right). Color code as in Fig. 1a. Note that inheritance of the sole paternal centriole by ABa-like is represented here. b) DIC images from a time-lapse recording corresponding to the stages schematized in (a), with an indication of the resulting AB-like, ABp-like, EMS-like, and P_2_-like blastomeres (at time 89:50; time in min:s since the beginning of the recording). See also corresponding Supplementary Video 1. c) TagRFP-T::SAS-7 (left, red) and SAS-4::GFP (right, cyan) live imaging of embryo shown in (b) (at time 89:50). The arrow points to the sole sperm-contributed centriole, which is present in the ABa-like blastomere in this particular embryo. A single optical slice is shown for SAS-4::GFP. Small boxes indicate magnified insets shown on the bottom left. d and e) Corresponding distribution in 4-cell embryos of sole sperm-contributed centriole marked by SAS-4::GFP; panel (d) reports the sum of sperm-contributed centrioles in AB-derived and P_1_-derived blastomeres and panel (e) their presence in AB-like, ABp-like, EMS-like, or P_2_-like blastomeres. Overall occurrences: ABa-like, 9; ABp-like, 6; EMS-like, 5; P_2_-like, 10. See Supplementary Table 1 for statistical analysis.

In this modified marked mating experiments, we shifted *zyg-1*(*b1ts*) mutant males from 15°C to 25°C during the third larval instar stage. This enables assembly of procentrioles in the proliferating mitotic zone of the gonad prior to the temperature shift but prevents their formation thereafter. As a result, most sperm cells derived from such animals contain a single centriole, which corresponds to the older one in the wild type, whereas the centriole that should have been generated next is missing (O’Connell *et al*. 2001). During the first cell cycle, this single sperm-contributed centriole seeds the formation of a procentriole in its vicinity. Because this centriole/procentriole pair remains joined until the end of the first cell cycle, a monopolar spindle assembles, leading to aberrant chromosome segregation and cleavage failure (Fig. 3a, left-most). In the second cell cycle, each centriole seeds the formation of a procentriole, leading to bipolar spindle assembly (Fig. 3a, second panel), and the generation of AB-like and P_1_-like cells in the third cell cycle (Fig. 3a, third panel). These 2 blastomeres then divide asynchronously, with the AB-like cell undergoing mitosis before the P_1_-like cell, as is the case for AB and P_1_ in the second cell cycle of wild-type embryos, ultimately yielding embryos that harbor AB-like, ABp-like, EMS-like, and P_2_-like blastomeres (Fig. 3a, right-most).

Using live imaging of the entire sequence of events to ensure proper staging of embryos (Fig. 3b and c; Supplementary Video 1), we addressed whether the single centriole contributed by sperm from *zyg-1*(*b1ts*) mutant males and harboring SAS-4::GFP segregates strictly to AB-derived or P_1_-derived cells, scoring blastomeres at the 4-cell stage. As shown in Fig. 3d and e, we found this not to be the case, as this single marked centriole can be present in either ABa-like, ABp-like, EMS-like, or P_2_-like blastomere (Supplementary Table 1). Although we cannot formally exclude that the aberrant divisions in this experimental settings might alter the distribution of the sole centriole, our findings indicate that the older sperm-contributed centriole is also distributed in a stochastic fashion in 4-cell stage embryos.

## Discussion

Our findings demonstrate that, in contrast to the stereotypy of centriole distribution during some asymmetric divisions in flies and mammals, sperm-contributed centrioles are distributed in a stochastic manner in embryos of *C. elegans*. It could have been envisaged that sperm-contributed centrioles are excluded from certain cells at the 4-cell stage, for instance the germ line precursor P_2_, thus ensuring that only newly made centrioles are transmitted in this lineage. Our work demonstrates that this is not the case. Therefore, although sperm-contributed centrioles persist for several hours during *C. elegans* embryogenesis (Balestra *et al*. 2015), the present findings establish that they cannot endow specific blastomeres in a stereotyped fashion with potential trans-generational biological information. Regardless, it will be interesting to address whether sperm-derived centrioles exhibit preferential inheritance at later stages of development. In this context, we note that experiments with Dendra2::SAS-4 showed that the older centriole marked following photo-conversion can also segregate to either daughter cell of ABprpppaa and ABprpppap (Erpf and Mikeladze-Dvali 2020), indicating that stochastic inheritance in the worm is not restricted to sperm-contributed centrioles. It will also be interesting to investigate whether the random distribution of sperm-contributed centrioles uncovered here is characteristic of worms, especially considering that nematode sperm is not flagellated, in contrast to the situation in most other metazoan species. Moreover, whereas the 2 *C. elegans* sperm-contributed centrioles are analogous at the ultrastructural level (Wolf *et al*. 1978), they are different from one another in other species, including *Drosophila* and man (Fawcett 1975; Blachon *et al*. 2009). Therefore, it is plausible that in those cases, such ultrastructural differences translate into stereotyped inheritance of sperm-contributed centrioles in the resulting embryos. Alternatively, the stochastic distribution revealed in our study might reflect a more general feature of the centriole organelle as it stands prior to fertilization.

## Materials and methods

### *C. elegans* strains

Worms were maintained using standard protocols (Brenner 1974). Strains of the following genotypes were utilized: *sas-7*(*Is1[tagRFP-T::sas-7* + *loxP]*) III, *fem-1*(*hc17ts*) IV (Doniach and Hodgkin 1984; Klinkert *et al*. 2019); *sas-7*(*or1940*[*gfp::sas-7*]) III (Sugioka *et al*. 2017); *sas-4*(*bs195[sas-4::gfp*]) III (a kind gift of Jessica Feldman and Kevin O’Connell); and *zyg-1*(*b1ts*) II, *sas-4*(*bs195*[*sas-4::gfp*] III (Wood *et al*. 1980). Strains carrying temperature-sensitive alleles were maintained at 15°C, other strains at 24°C. To obtain hermaphrodites lacking sperm, *sas-7*(*Is1[tagRFP-T::sas-7* + *loxP]*) and *fem-1*(*hc17ts*) were allowed to lay embryos overnight at 15°C on a plate, which was then shifted to 25°C for ∼36–48 h. The resulting feminized, sperm-less, hermaphrodites were mated to *sas-4*(*bs195[sas-4::gfp]*) or *sas-7*(*or1940[gfp::sas-7]*) males and their progeny analyzed by live imaging or following fixation (see below). To obtain sperm cells with a single centriole marked with SAS-4::GFP, *zyg-1*(*b1ts*), *sas-4*(*bs195[sas-4::gfp]*) males were grown initially at 15°C and shifted as L3 larvae to 25°C for ∼24–48 h prior to mating. Most resulting sperm harbored a single centriole, although sperm with 2 or 0 centrioles were observed occasionally.

## Live imaging and analysis

Embryos were dissected from the uterus of hermaphrodites in a watch glass containing M9 and transferred using a mouth pipette onto a 2% agarose pad, which was overlaid gently with an 18 × 18 mm coverslip. Time-lapse DIC and dual color fluorescent microscopy imaging was performed at ∼21°C on a Zeiss Axioplan 2 with a 63 × 1.40 NA lens, with binning 2 and a 6% neutral density filter to attenuate the 120W arc mercury epifluorescent source. The motorized filter wheel, external shutters, and the 1,392 × 1,040 pixels 12-bit Photometrics CoolSNAP ES2 camera were controlled by µManager (www.micro-manager.org). Typically, a *z*-stack of 15 planes 1 *µ*m apart was taken at the 4-cell stage, with exposure times of 50 (DIC), 100 (GFP), and 100 ms (TagRFP-T). For display (Fig. 2a–e; Supplementary Fig. 1), planes with centriolar signal were retained and max intensity projected in Fiji. Brightness and contrast were adjusted slightly for better visualization, using identical settings within a series.

## Indirect immunofluorescence and confocal microscopy

Methanol fixation was performed essentially as described (Gönczy *et al*. 1999). In brief, gravid hermaphrodites were dissected on polylysine-coated slides, covered with a 12 × 12 mm coverslip, and the slide frozen on a metal block precooled on dry ice. After a few minutes, embryos were freeze-cracked and fixed in −20°C methanol for ∼3 min. Following 2 PBS washes, slides were incubated 45–60 min at room temperature with primary antibodies in PBT (PBS + 0.05% Tween-20). Primary antibodies were 1:50 mouse anti-IFA (Leung *et al*. 1999) and 1:500 rabbit anti-GFP (a kind gift of Viesturs Simanis). After 2 PBS washes, slides were incubated 45–60 min at room temperature with secondary antibodies in PBT (1:500 goat anti-rabbit-Alexa488 and goat anti-mouse-Alexa568, Thermo Fisher Scientific). Slides were counterstained with 1 *µ*g/ml Hoechst 33258 (Sigma) to reveal DNA and washed twice with PBS. Thereafter, 7 *µ*l of mounting medium (0.189 mol/l n-propyl gallate, 90% glycerol, 10% PBS) was pipetted onto the specimen, which was covered with an 18 × 18 mm coverslip, applying slight pressure to remove excess liquid prior to analysis by wide-field microscopy. Indirect immunofluorescence was imaged on an upright LSM700 Zeiss confocal microscope with a Plan-Apochromat 63 × 1.4 NA lens, collecting 1,024 × 1,024 pixels optical slices 0.35 *µ*m apart, using 405-, 488-, and 555-nm solid state lasers. Relevant planes containing centriolar signals were max intensity projected in Fiji. Brightness and contrast were adjusted slightly for better visualization, using identical settings within a series.

## Ethanol fixation

Gravid hermaphrodites were collected from plates with ∼5-ml M9, spun at 1,500 rpm for 2 min in a clinical centrifuge, followed by 2 washes with M9. The supernatant was removed and 1.5-ml 100% ethanol then added. Approximately 1 min thereafter, ethanol was removed and the worm pellet resuspended in 15-*µ*l MVD (50% M9, 50% Vectashield (Vector), 0.7 *µ*g/L Hoechst 33258). After rehydration for 1 min or more, worms were transferred onto a slide and covered with a 20 × 40 mm coverslip, applying slight pressure to remove excess liquid prior to analysis of fixed embryos inside the uterus.

## Statistics

The presence of sperm-contributed centrioles in a given blastomere was determined and corresponding contingency tables generated using the Python library pandas (https://pandas.pydata.org/about/citing.html). We then tested whether sister cells (ABa vs ABp, and EMS vs P_2_) or any of the 4 possible configurations at the 4-cell stage (ABa and P_2_, ABa and EMS, ABp and P_2_, ABp and EMS) were more likely to harbor sperm-contributed centrioles using the chi-square test as implemented in the library SciPy (https://scipy.org/citing-scipy/). An analogous analysis was conducted with embryos that inherited a single sperm-contributed centriole. See Supplementary Tables 1 and 2 for details.

## Supplementary Material

iyad048_Supplementary_Data

## Data Availability

The authors affirm that all the data necessary for confirming the conclusions of the article are present within the article, figures, and tables. Strains are available upon request. Supplemental material is available at GENETICS online.
