# Supplementary material for: Sperm-contributed centrioles segregate stochastically into blastomeres of 4-cell stage Caenorhabditis elegans embryos
Source: Genetics. 2023 Mar 29;224(1):iyad048. doi: 10.1093/genetics/iyad048 (PMC10158834; doi:10.1093/genetics/iyad048)
Supplement: iyad048_Supplementary_Data [file iyad048_supplementary_data.zip › Supplemental_Figures_GENETICS-2023-306003.pdf]

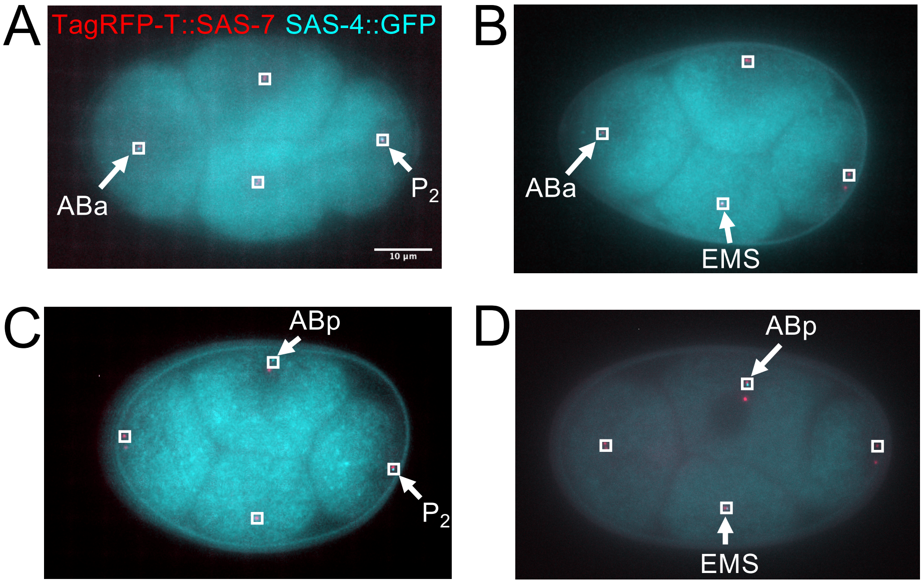

Figure S1

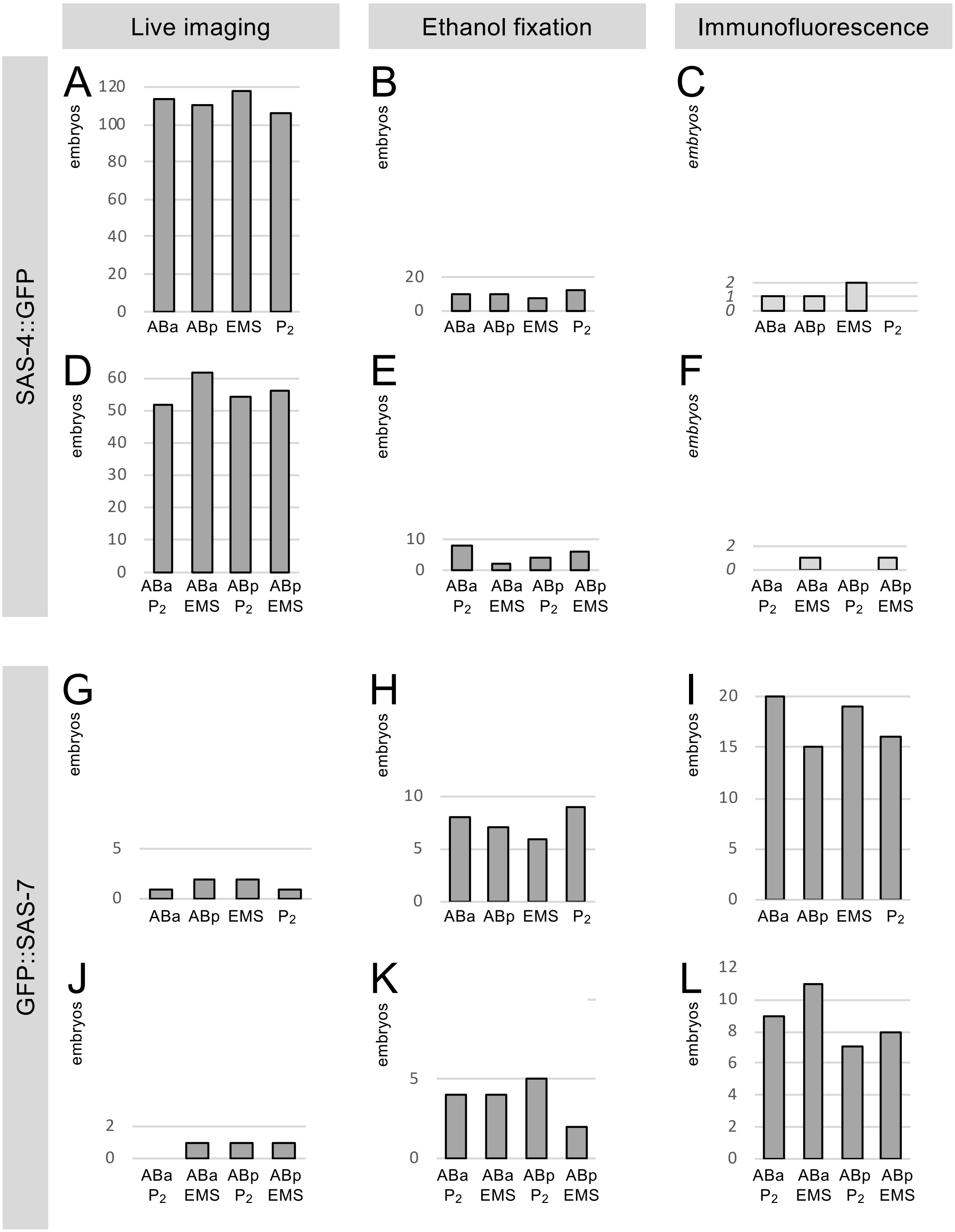
Live imaging
Ethanol fixation
Immunofluorescence
SAS-4::GFP
GFP::SAS-7
A
B
C
D
E
F
G
H
I
J
K
L
embryos
ABa
ABp
EMS
P2

Figure S2

# sperm-contributed centrioles	figure	marker	comparison	n (embryos)	chisquare	p value
2	Fig. 2F	GFP::SAS-4	ABa vs ABp	246	0.065	0.799
2	Fig. 2F	GFP::SAS-4	EMS vs P_2	246	0.407	0.524
2	Fig. 2G	GFP::SAS-4	All equal?	246	0.472	0.925
2	Fig. 2J	GFP::SAS-7	ABa vs ABp	53	0.472	0.492
2	Fig. 2J	GFP::SAS-7	EMS vs P_2	53	0.019	0.891
2	Fig. 2K	GFP::SAS-7	All equal?	53	0.962	0.81
1	Fig. 3E	GFP::SAS-4	All equal?	30	2.267	0.519
1	Fig. 3E	GFP::SAS-4	ABa vs ABp	15	0.6	0.439
1	Fig. 3E	GFP::SAS-4	EMS vs P_2	15	1.667	0.197

Table S1

# sperm-contributed centrioles	figure	marker	imaging	comparison	n (embryos)	chisquare	p value
2	Fig. S2A	GFP::SAS-4	Live imaging	ABa vs ABp	224	0.071	0.789
2	Fig. S2A	GFP::SAS-4	Live imaging	EMS vs P_2	224	0.643	0.423
2	Fig. S2D	GFP::SAS-4	Live imaging	All equal?	224	1	0.801
2	Fig. S2I	GFP::SAS-7	IF	ABa vs ABp	35	0.714	0.398
2	Fig. S2I	GFP::SAS-7	IF	EMS vs P_2	35	0.257	0.612
2	Fig. S2L	GFP::SAS-7	IF	All equal?	35	1	0.801

Table S2
