# Supplementary material for: Sperm-contributed centrioles segregate stochastically into blastomeres of 4-cell stage Caenorhabditis elegans embryos
Source: Genetics. 2023 Mar 29;224(1):iyad048. doi: 10.1093/genetics/iyad048 (PMC10158834; doi:10.1093/genetics/iyad048)
Supplement: iyad048_Supplementary_Data [file iyad048_supplementary_data.zip › Supplemental_Material_GENETICS-2023-306003.docx]

**Supplemental Figure Legends**

**Figure S1. Four classes of sperm-contributed centriole distributions at the 4-cell stage**

**(A-D)** Merged TagRFP-T::SAS-7 (red) and SAS-4::GFP (cyan) live imaging of 4-cell stage embryo resulting from the mating of TagRFP-T::SAS-7 *fem-1*(*hc17ts*) hermaphrodites with males expressing SAS-4::GFP. In each embryo, arrows point to the two blastomeres that inherited the sperm-contributed centrioles and insets correspond to the magnified views in Figure 2B-E (A: ABa and P_2_, same embryo as in Fig. 2A, B: ABa and EMS; C: ABp and P_2_; D: ABp and EMS).

**Figure S2.** **Distribution of sperm-contributed centrioles in 4-cell stage embryos by experimental type**

Distribution of sperm-contributed centrioles marked by SAS-4::GFP (A-F) or GFP::SAS-7 (G-L) in 4-cell stage embryos by experimental type (A, D, G, J: live imaging; B, E, H, K: ethanol fixation; C, F, I, L: immunofluorescence). Occurrences (ABa, ABp, EMS, P_2_): 114, 110, 118, 106 (A); 10, 10, 8, 12 (B); 1, 1, 2, 0 (C); 1, 2, 2, 1 (G); 8, 7, 6, 9 (H); 20, 15, 19, 16 (I). Occurrences (ABa and P_2_, ABa and EMS, ABp and P_2_, ABp and EMS): 52, 62, 54, 56 (D); 8, 2, 4, 6 (E); 0, 1, 0, 1 (F); 0, 1, 1, 1 (J); 4, 4, 5, 2 (K); 9, 11, 7, 8 (L). Note that the scale of the Y axis is different in C compared to A and B, as well as in F compared to D and E, as indicated also by the italics.

**Video S1. Sequence of events in embryos derived from fertilization by sperm harboring a single centriole**

DIC time-lapse recording of embryo derived from mating TagRFP-T::SAS-7 *fem-1*(*hc17ts*) hermaphrodites with *zyg-1*(*b1ts*) mutant males expressing SAS-4::GFP (not shown) and shifted during spermatogenesis to 25^o^C, yielding sperm with only one, older, centriole. Elapsed time since the beginning of the recording is shown in min:sec. The embryo is ~50 μm-long; anterior is to the left.

**Table S1. Statistical analysis -main figures**

Chi-square test and corresponding p values of data reported in the indicated panels of Fig. 2 and Fig. 3.

**Table S2. Further statistical analysis -supplementary figure S2**

Chi-square test and corresponding p values of data reported in the indicated panels of supplementary figure S2 (in each case: principal analysis modality).
